# A Review on Reverse Osmosis and Nanofiltration Membranes for Water Purification

**DOI:** 10.3390/polym11081252

**Published:** 2019-07-29

**Authors:** Zi Yang, Yi Zhou, Zhiyuan Feng, Xiaobo Rui, Tong Zhang, Zhien Zhang

**Affiliations:** 1 Department of Materials Science and Engineering, The Ohio State University, 2041 N. College Road, Columbus, OH 43210, USA; 2 State Key Laboratory of Precision Measurement Technology and Instrument, Tianjin University, Tianjin 300072, China; 3 Institute for Advanced Materials and Technology, University of Science and Technology Beijing, Beijing 100083, China; 4 William G. Lowrie Department of Chemical and Biomolecular Engineering, The Ohio State University, Columbus, OH 43210, USA

**Keywords:** ceramic membranes, polymeric membranes, reverse osmosis, nanofiltration, water purification, desalination

## Abstract

Sustainable and affordable supply of clean, safe, and adequate water is one of the most challenging issues facing the world. Membrane separation technology is one of the most cost-effective and widely applied technologies for water purification. Polymeric membranes such as cellulose-based (CA) membranes and thin-film composite (TFC) membranes have dominated the industry since 1980. Although further development of polymeric membranes for better performance is laborious, the research findings and sustained progress in inorganic membrane development have grown fast and solve some remaining problems. In addition to conventional ceramic metal oxide membranes, membranes prepared by graphene oxide (GO), carbon nanotubes (CNTs), and mixed matrix materials (MMMs) have attracted enormous attention due to their desirable properties such as tunable pore structure, excellent chemical, mechanical, and thermal tolerance, good salt rejection and/or high water permeability. This review provides insight into synthesis approaches and structural properties of recent reverse osmosis (RO) and nanofiltration (NF) membranes which are used to retain dissolved species such as heavy metals, electrolytes, and inorganic salts in various aqueous solutions. A specific focus has been placed on introducing and comparing water purification performance of different classes of polymeric and ceramic membranes in related water treatment industries. Furthermore, the development challenges and research opportunities of organic and inorganic membranes are discussed and the further perspectives are analyzed.

## 1. Introduction

**Figure 1:**
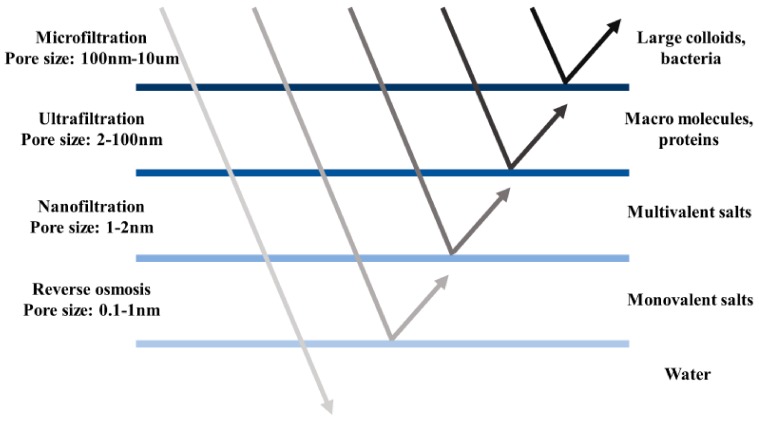
Classification of membranes for water purification in terms of pore size and retained species.

Human welfare has been promoted by continued economic growth, which is accounted for by mechanization and industrialization. However, increasing income and wealth would cause ecological problems, since natural resources are used as inputs of several products, and the pollution of the environment is directly linked to the production scale [1,2]. Water shortage is one of the problems caused by global industrialization. In developing countries, untreated wastewater entered rivers and seas, leading to ground water contamination and limited clean water supply. In order to protect the environment and save mankind, various actions have been taken to tackle industrial pollutants [3,4,5,6,7]. On the other hand, continued population expansion and urbanization also lead to increasing residential water demand. The United Nations predicts that with the current population growth rate, in ten years half of the geographic regions of the world will be impacted by water scarcity [8]. Water purification and desalination have been used more and more around the world to provide people with fresh and clean water, especially in water-stressed countries such as Qatar, the United Arab Emirates, and Israel. These regions need inventive and viable approaches for safe water supply to support population growth. Since 1980, filtration systems equipped with nanoporous membranes have been commercialized and membrane separation has become a rapidly emerging technology in many industrial applications such as food industry, petroleum industry, chemical processing industry, pulp and paper industry, pharmaceuticals and electronic industry [9,10,11,12,13,14]. In these industries, wastewater purification is an essential process that involves membrane separation technique. According to particle size of retained species, water purification systems such as reverse osmosis (RO), nanofiltration (NF), ultrafiltration (UF) and microfiltration (MF) have been introduced globally [15,16,17,18]. A description of membrane types with corresponding pore diameter and retained species is shown in Figure 1. Meanwhile, significant progress has been made in research on RO membranes made from different materials for desalination applications [19].

**Figure 2:**
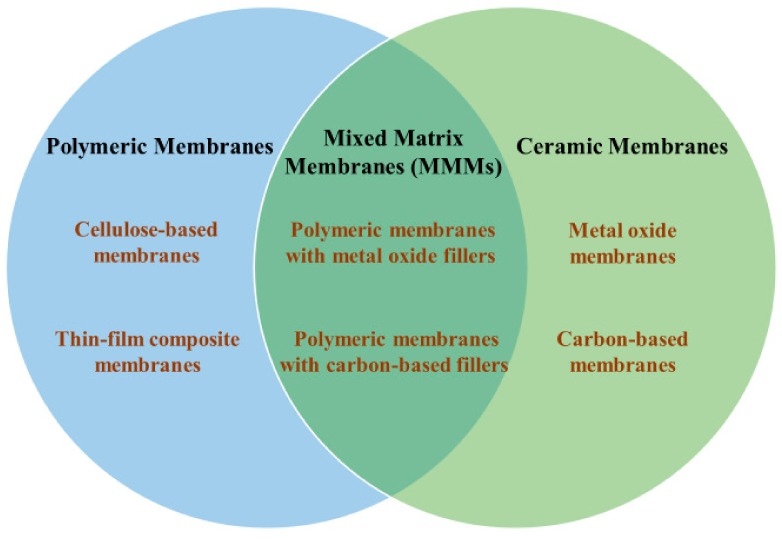
Representative reverse osmosis (RO) and nanofiltration (NF) membranes for water treatment.

It is well known that polymeric membranes are currently used the most in seawater desalination and wastewater treatment industries due to their well-developed and outstanding performance [20,21,22]. Research is still being conducted to solve problems related to performance limitations and post-treatment process. Fouling is one of the main drawbacks of polymeric membranes. Surface structure and materials have been modified to suppress fouling effect. Introduction of materials that contain inorganic fillers in organic matrix such as mixed matrix membranes (MMMs) is a significant achievement for underlying issues. In addition to slow improvement achieved in polymeric membranes, inorganic membranes have gained growing interest due to their long-term chemical and thermal stabilities and high mechanical strength [23]. In general, inorganic membranes include metal oxide membranes and carbon-based membranes (Figure 2). Alumina, zirconia, titania and their mixtures are the most commercialized metal oxide membranes in the market. Almost all inorganic membranes share a common structure, containing a macro-porous support and a meso- or micro-porous barrier layer. In the industry, ceramic membranes are usually used in systems whose operating conditions are challenging to polymeric membranes (high temperature, corrosive effluent, etc.). However, recent studies on cost-effective preparation method using cheap materials indicate a commercialization potential for ceramic membranes [24,25]. In addition, ceramic membranes synthesized from advanced porous materials such as carbon nanotubes (CNTs) and graphene oxide (GO) have been identified as the most promising inorganic membranes in thin film technology [26,27]. These membranes have excellent permeability and selectivity, and their structures offer high productivity and practically efficient performance in desalination and water purification processes.

This paper critically reviews the growth and achievement in organic and inorganic membrane studies for RO and NF procedures. The review will start by introducing the synthesis method and structural properties of recent RO and NF membranes, followed by discussing and comparing water purification performance of representative RO and NF membranes made from organic and inorganic materials. The wide scope of this review highlights the potential of RO and NF membranes made from new materials for further research and improvement. Finally, challenges and remaining issues that need to be addressed for further work are summarized.

## 2. Reverse Osmosis and Nanofiltration Membranes

### 2.1. Polymeric Membranes

Polymeric/organic RO and NF membranes have dominated the global market since 1980 due to their excellent performance and low cost. Some state-of-the-art polymeric RO and NF membranes are listed in Table 1 together with manufacturer, selective layer composition, operation condition, and purification performance. It can be seen that current market is dominated by thin-film composite (TFC) membranes due to their outstanding performance. Important polymers that are being used for making RO and NF membranes are polyamides, cellulose acetate, cellulose diacetate, cellulose triacetate, piperazine, etc. Polyamide is a macromolecule containing recurring amide (-CO-NH-) groups, and can be found both naturally and artificially. Examples of natural polyamide are wool, silk, and angora. Cellulose-based polymers are usually prepared by phase inversion method, as introduced in Section 2.1.1. In this section, two classes of organic membranes made from different polymeric materials are reviewed.

#### 2.1.1. Cellulose-Based Membranes

Cellulose-base (CA) membranes have been developed and commercialized for more than 60 years. In 1955, cellulose acetate membranes were prepared and introduced by Reid et al. using acetone as the solvent [28]. The general synthesis process of CA membrane is called phase inversion method: cellulose triacetate is first dissolved in an organic solvent or solvent mixture to form a casting solution. Then the solution is coated on a flat or tubular support. Finally, the support is immersed in a non-solvent bath, where polymer coagulation occurs and a CA membrane forms. Although CA membranes made by Reid at al. had good selectivity, the water permeability was extremely low and could not be used for practical applications. In 1963, Loeb et al. invented the first efficient RO membrane: cellulose diacetate (CDA) membrane. CDA membranes had much higher flux compared to CA membranes but were prone to biological attack [29]. The invention of CDA membranes accelerated the development of cellulose triacetate (CTA) membranes, which had slightly stronger thermal, chemical, and biological stabilities [30]. With asymmetric morphologies, cellulose-based membranes have anisotropic structures, consisting of an upper skin layer on a porous sublayer [31]. Both the skin layer and porous sublayer have identical chemical composition. The filtration performance of CA membranes depends on the degree of acetylation. For instance, CA membrane with 40 wt% acetate and a 2.7 degree of acetylation had a salt rejection between 98% and 99% [32]. Higher acetylation will result in higher selectivity but lower water permeability. CA membranes are stable in pH range 4–6. In acidic and basic feed solutions, hydrolysis reaction will happen and lower the selectivity.

Though membranes with better separation performances and comparable costs were fabricated, some studies were reported to improve CA membranes. Chou et at. found dispersing silver nanoparticles on CA membrane surface would increase its biological stability while maintain the permeability and salt rejection [33]. Coating phospholipid polymer on CA membrane during phase conversion resulted in a fouling-resistant membrane with high water flux [34]. A small percentage of mineral fillers such as aluminum oxide improved the compaction resistance of CA membranes remarkably [35]. During the past four decades, thin-film composite (TFC) membranes, whose permeability and rejections surpass those of CA membranes, have dominated the market. However, CA membrane still exists due to its overall exceptional chlorine resistance, which depends on several parameters such as polymer type, synthesis procedure, and pH of feed solution. Since feed water disinfection is a necessary step in RO and NF installations and chlorine is the most common choice of disinfectants, it is important to have chlorine-tolerant membranes for water treatment. Table 2 shows effects of various processing methods on chlorine resistance. Current research mainly focuses on modifications of TFC membranes for chlorine resistance improvement.

#### 2.1.2. Thin-Film Composite Membranes

**Figure 3:**
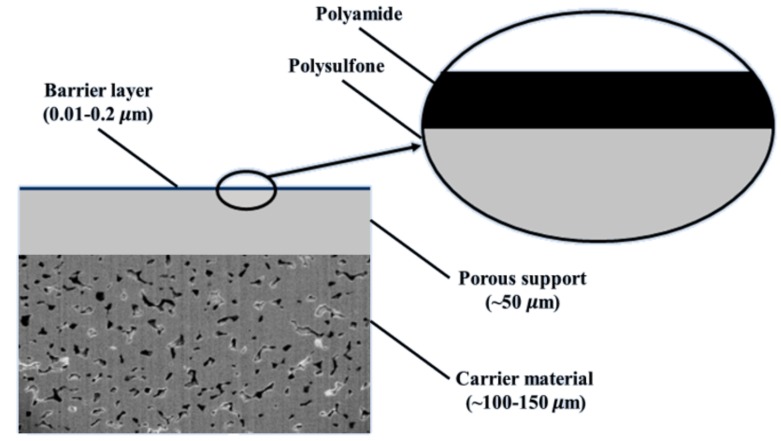
Thin-film composite membrane structure.

TFC membranes were invented by Cadotte in the 1970s, but were not widely used until the second half of the 1980s [43]. Polyamide (PA) membranes were developed by Hoehn and Richter and had good water purification performance. The main drawback of PA membranes was susceptibility to free chlorine attack [44]. After development of TFC membranes, it was found the PA TFC membranes had outstanding separation performance as well as better chlorine resistance. As shown in Figure 3, the structure of a PA TFC membrane consists of a thin selective barrier layer on a porous support [45,46,47]. The support has a microporous structure (UF membrane), providing mechanical strength and high water flux, and the barrier layer has a function of ion separation. Compared with CA membranes, which can only be made from linear, soluble polymers, TFC membranes have more desirable characteristics. Many materials (linear and crosslinked polymers) and approaches can be used to synthesize or modify the porous support and barrier layer individually to optimize the thermal and chemical stabilities, permeability, salt rejections, etc. Many papers focus on improving TFC membranes for RO applications have been published. On the other hand, the manufacturing cost of TFC membranes is higher than that of CA membranes since at least two membrane fabrication steps are needed: synthesis of microporous support followed by synthesis and deposition of barrier layer on microporous support.

The porous support plays an important role in providing mechanical strength to withstand high pressure during RO and NF processes. Meanwhile to form a defect-free barrier layer, the surface of the support needs to be uniform and smooth. Polysulfone is one of the most significant microporous supports for TFC membranes [48]. The surface pore size of polysulfone support ranges from 1.9 nm to 15 nm, with a surface porosity up to 16% [49,50]. The selectivity generally increases with decreasing pore size [51]. Since polysulfone shows good structural stability in a wide pH range, barrier layers made from highly acidic or alkaline precursors can be coated on polysulfone substrates. The disadvantages of polysulfone include poor weatherability, low chlorine resistance, and prone to stress cracks. Adding nanoparticles and applying new preparation methods are two main approaches to improve polysulfone supports. A chlorine-resistant TFC membrane can be made by metalation sulfochlorination of polysulfone [52]. Plasma treatment on polysulfone support results in the exhibition of hydrophobicity, which optimizes chlorine resistance and water permeability [53,54]. In addition to polysulfone, CA, polyimide, polypropylene, polyketone and polyethylene terephthalate (PET) have also been used as porous supports [55,56,57,58]. A hydrolyzed PA CA membrane has been fabricated and the covalent bond between porous CA support and selective PA barrier layer indicates a chemical stable structure. This membrane exhibits a NaCl rejection up to 97% [58]. In addition, TFC membranes synthesized by heat and plasma treatments using electrospun nanofibers as supports showed remarkable filtration performance [59]. Yoon et al. have prepared a PA TFC membrane using polyacrylonitrile (PAN) nanofibrous scaffold as porous support. The experimental result showed the PA PAN composite membrane has similar sulfate rejection rate (98%) but 38% higher water permeability compared to commercial NF membranes (NF270) [60]. Several recent studies focus on the effect of support pore size on barrier layer formation and water purification performance, but there have been no consistent conclusions so far [61,62].

**Figure 4:**
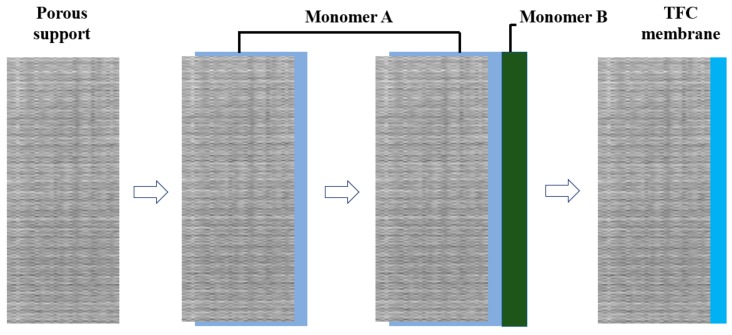
Mechanism of interfacial polymerization.

Most selective barriers of TFC membranes are synthesized by interfacial polymerization, which occurs at an interface between two immiscible monomers/solvents [63,64]. Once a layer forms at the interface, solvents from both sides cannot pass through it and therefore the reaction stops, producing a membrane thinner than 200 nm (Figure 4). Heat treatment is necessary since interfacial polymerization happens at elevated temperature. The purification performance of TFC membranes is primarily determined by barrier layer, which is affected by solvent type and concentration, curing condition and temperature. Table 3 summarizes precursors for preparing TFC membranes by interfacial polymerization method for water purification in recent studies. Due to their good mechanical property and outstanding rejection ratio, TFC membranes are used in a large number of purification tasks, especially in desalination. The main problem associated with TFC membranes is their flux and salt rejection decrease gradually as a result of fouling, particularly in treating with wastewater containing bacteria and nutrients. According to Mansourpanah et al., TFC membranes with antifouling property can be prepared by grafting functional groups or adding hydrophilic additives on membrane surface through radiation or plasma treatment [65]. The altered barrier layer becomes smooth, hydrophilic and has similar surface charge as foulants. Therefore the interaction between contaminants and membrane surface is reduced. It is also found that TFC membranes blended with polyacrylamide and polymethacrylic acid exhibit biofouling resistance [66]. Deposition of natural hydrophilic polymers such as sericin would increase surface hydrophilicity of TFC membranes, and improves selectivity and fouling resistance [67]. Another drawback of TFC membranes is poor chlorine resistance. During water purification process, chlorine (frequently used as disinfectant) changes the hydrogen bounding in TFC membranes, resulting in performance decay [68]. Thus, it is essential to increase chlorine resistance of TFC membranes. A chlorine-resistant TFC membrane has been invented by Yao et al. by secondary interfacial polymerization method to eliminate the interaction between unreacted amino groups and free chlorine [69]. Experimental results indicated TFC membranes blended with layered double hydroxides (LDHs) have high porosity and hydrophilicity, exhibiting superior chlorine resistance and anti-fouling capacity [70]. Similar studies focus on enhancing chlorine resistance of TFC membranes by incorporating additives are available in literature [71,72,73]. From a technique perspective, methods such as atomic layer deposition (ALD) controls membrane thickness precisely through sequential surface reactions [74]. Hydrophilic selective barriers synthesized using this technology have excellent fouling and chlorine resistance.

### 2.2. Ceramic Membranes

Although ceramic/inorganic RO and NF membranes have only been studied for 30 years and are in early stage of commercialization, their encouraging performance, as exemplified in Table 4, offers great potential for water purification. In this section, two classes of ceramic membranes made from different inorganic materials are discussed.

#### 2.2.1. Metal Oxide Membranes

Compared to polymeric membranes, inorganic membranes offer higher chemical stability and stronger mechanical properties. Metal oxides such as alumina, zirconia, and titania form an important class of ceramic membranes. Conventionally, a RO metal oxide membrane has an asymmetric structure consisting of a thick macroporous (>50 nm) support, an intermediate mesoporous (2–5 nm) layer, and a thin selective (<1 nm) top layer. A NF metal oxide membrane has similar structure as RO metal oxide membrane but contains no selective top layer [101,102,103]. The most widely used approach for preparing metal oxide ceramic membranes is sol-gel method, which converts precursor solutions into solid membranes in four steps: precipitation reaction first happens between hydrolyzed precursors, followed by a peptization reaction in which precipitation transforms into a colloid sol. The stable sol is then coated on a porous support and gelates during drying. Finally high temperature sintering is applied to the membrane to optimize mechanical properties and pore structure [8,89]. In order to make homogeneous membranes with less defects, colloidal particles are dispersed uniformly in the solvent by stabilizers such as nitric acid, ethanolamine (MEA) and triethylenetetramine (TETA) [104,105,106]. Since complex fabrication process of multi-layered membranes as well as expensive precursor materials indicating high manufacturing cost, simplified synthesis method and use of cheap materials will reduce the production cost and accelerate the development and commercialization of ceramic membranes.

One of the most widely studied inorganic membranes is alumina membrane, which has an average pore size of 2–5 nm (MWCO of 3000–1000 Da) and is commonly used in NF systems or as an intermediate layer in RO membranes [107]. Alumina membranes with pore size smaller than 1 nm has been made, but showed low permeability (5 LMH/bar) and cannot be used for industrial purposes [8]. Wang el al. have prepared a supported γ-Al_2_O_3_ hollow fiber membrane with a mean pore size of 1.61 nm that demonstrates a high water permeability of 17.4 LMH/bar [85]. This membrane exhibits good selectivity for multivalent ions such as Ca^2+^ (84.1%), Mg^2+^ (85%), Al^3+^ (90.9%) and Fe^3+^ (97.1%), but very low retention of monovalent ions such as NH_4_^+^ (27.3%) and Na^+^ (30.7%). Recent studies focus on surface modification of alumina membrane to further improve its purification performance. For instance, a mixed matrix carbon molecular sieve (CMS) and α-Al_2_O_3_ membrane fabricated by vacuum-assisted impregnation method has a water flux up to 25 kg m^−2^ h^−1^ and a salt rejection between 93% and 99% when tested using 3.5 wt% NaCl (seawater) at 75 °C [87]. Ren et al. changed the surface of a porous alumina membrane from hydrophilic to hydrophobic by fluoroalkylsilane (FAS) grafting, resulting in a water flux of 19.1 LMH and salt rejection over 99.5% [88]. Such outstanding salt retention and water permeability hold promise for practical desalination applications. In addition to surface modification, using cheap precursor materials provides both economic and environmental benefits. Researchers have used Al_2_O_3_ hollow fiber supports and coal fly ash, a byproduct of coal burning, to synthesize Al_2_O_3_-NaA zeolite membranes successfully. The Al_2_O_3_-NaA zeolite membrane has been used to treat wastewater containing lead ions (Pb(Ⅱ), 50 mg L^−1^) and possesses a Pb(Ⅱ) removal rate of 99.9% [108].

Zirconia and titania are other popular materials for ceramic membranes. In sol-gel method, zirconium alkoxides are often used as precursors to prepare zirconia sols [109,110]. However, some zirconium alkoxides such as zirconium propoxide is water-reactive, which could produce agglomerates rather than stable nanoparticles. Therefore at the beginning few laboratories had successfully synthesized zirconia membranes. In 1998, Garem et al. discovered that adding 13 mol% magnesium would enhance the chemical and thermal stabilities of zirconia sols [111]. Since then many stabilizers have been investigated for preparing zirconia membranes. Glycerol has been introduced into the sol-gel process to make ZrO_2_ NF membranes for treating high-salinity wastewater. More specifically, glycerol binds to the surface of ZrO_2_ nanoparticles as a capping agent and prevents phase transformation during calcination. The crack-free ZrO_2_ NF membrane exhibits a permeability of 13 LMH/bar and approximately 68% rejection rate when filtering NaCl solutions with mass fraction up to 24.92% [90]. Lu et al. have used zirconium salts and titanium alkoxides as sol-gel precursors to prepare a TiO_2_-doped ZiO_2_ NF membrane [91]. The addition of Ti^4+^ suppresses zirconia phase transformation, narrows the pore size distribution and increases the specific surface area. This membrane has high water permeability above 35 LMH/bar with a MWCO of 500 Da, and simulated retention rates of 99.6% for Co^2+^ and 99.2% for Sr^2+^, indicating its attractive potential for radioactive wastewater treatment. Compared with alumina and zirconia membranes, the surface pore size and phase composition of titania membranes can be controlled by synthesis procedure. Anatase is the most preferable crystal form of titania due to its exceptional stability and narrow pore size distribution. A TiO_2_ membrane with a pore diameter of 4 nm has been fabricated successfully by gentle heat treatment and remained stable in various solutions (brackish water, sea water and brine water) for over 350 h [89].

In addition to traditional metal oxide membranes, composite membranes made of two or more metal oxides is a current research focus. For example, a bilayer membrane containing a TiO_2_ layer on top of a ZnAl_2_O_4_ layer has been prepared and evaluated. It has been proved that compare to single layer membrane made from 50 mol% TiO_2_ and 50 mol% ZnAl_2_O_4_ with similar pore size, the bilayer membrane which has opposite surface charges could increase the electric interactions between membrane pores and filtered ions, and therefore produces a higher salt rejection, especially for divalent salts [112]. Another example of inorganic composite membranes is CoO-SiO_2_ membrane synthesized by Elma et al. for desalination applications [94]. The effects of cobalt addition (up to 35 mol%), feed solution concentration (0.3–7.5 wt% NaCl), and operation temperature (22–60 °C) on purification performance were investigated systematically. Experimental results showed the volume fraction of silica mesopores increases with cobalt concentration, and with over 99.7% NaCl retention rate at all times, the highest water flux of 20 kg m^−2^ h^−1^ was achieved for 0.3 wt% feed solution at 60 °C. Furthermore, a series of studies confirm that silica membranes blended with cobalt oxide exhibit not only excellent desalination performance but also robust structures compared to single-element SiO_2_ membranes [92,93].

In spite of prominent outcomes of metal oxide RO and NF membranes, certain shortcomings such as raw material cost and membrane thickness have hindered their commercialization for water purification. These issues can be overcome by further reducing the membrane thickness or exploring other cheap materials that have great chemical and thermal stabilities. Membranes that have strong surface charges in aqueous environment are also attractive.

#### 2.2.2. Carbon-Based Membranes

In recent years, ordered mesoporous materials (OMMs) have attracted increasingly research interests in addressing water pollution and water shortage problems [113,114]. Among all kinds of OMMs, ordered mesoporous carbons (OMCs) such as carbon nanotubes (CNTs) and graphene possess important properties including large specific surface area, highly uniform structure with tunable pore size and strong atomic bonds, thus have been selected as promising candidates for wastewater treatment applications [115,116,117]. As one of fullerene derivatives, CNTs are cylindrical molecules composed of rolled-up graphite sheets with diameter ranges from 1 nm to several centimeters [118]. Based on the layers of graphite sheets, CNTs can be further classified into single-walled carbon nanotubes (SWCNTs), double-walled carbon nanotubes (DWCNTs) and multi-walled carbon nanotubes (MWCNTs). For water desalination and purification applications, CNTs can be fabricated into standalone membranes or incorporated with other materials in many formats. An investigation of a highly stable and electrochemically active membrane made solely of CNTs, which could find significant applications in chemical and biological wastewater treatment, was undertaken by Sadia et al. [119]. Such CNTs membrane maintained a phenol removal rate over 85% for 4 h with an average oxidation rate of ~0.059 mol h^−1^ m^−2^ when operated with H_2_O_2_. Since water molecules can transport through CNTs structure without much impedance, some CNTs membranes used in RO systems with outstanding salt rejections as well as high water permeabilities have been reported [120,121,122]. On the other hand, the incorporation of CNTs into polymeric or inorganic matrix makes it possible to modify membrane properties and further improve surface hydrophilicity, fouling resistance, structural stability and salt retention. Yang et al. have confirmed a polyvinyl alcohol (PVA) based carboxylic MWCNTs membrane synthesized by interfacial adhesion method has better thermal stability and separation performance than a PVA membrane without carboxylic MWCNTs [123]. This PVA/C-MWCNT membrane exhibits a water flux of 6.96 kg m^−2^ h^−1^ and a NaCl rejection of 99.91% at 22 °C. In the work conducted by Peydayesh et al., hyperbranched polyethyleneimine modified MWCNTs were incorporated into polyethersulfone matrix to form a positively charged NF membrane, which had a average pore size of 0.81 nm and an enhanced water flux of 75.7 LMH [124]. The hybrid membrane showed superior retention rates for heavy metals (i.e., 99.06% for Zn^2+^, 94.63 for Ni^2+^, and 93.93% for Pb^2+^) and antifouling property due to effective membrane surface charge and hydrophilicity, respectively.

Despite advantages of CNTs, drawbacks such as high cost and low selectivity for certain ions (arsenate, arsenic, and sodium) have limited their commercialization [118]. Graphene, a cost-effective two-dimensional carbon allotrope that consists of a monolayer of carbon atoms arranged in hexagonal lattice, has been found to be a highly permeable and selective material for water purification processes [125,126]. Since water flux across a membrane is inversely proportional to the membrane thickness, single-atom-thick graphene offers an opportunity for exceptional permeability and efficient energy utilization [127]. Pure graphene has a closely packed structure which is impermeable to gas and liquid molecules. Therefore to improve permeability and ion selectivity defects or functional groups must be generated designedly. Nanoporous graphene can be fabricated either by electrochemical modification of pristine graphene or by growth on supports from different chemical reactions [128]. The most commonly applied techniques to generate nanosized pores on graphene structure include high-temperature oxidation, ultraviolet (UV) ozone treatment and plasma etching [129,130,131]. Sub-nanometer-sized pores on monolayer graphene have been created successfully for nanofiltration and desalination applications [132]. During synthesis process, small defects were first introduced by ion bombardment and further enlarged by oxidative etching. The experimental results revealed that the separation mechanisms of the porous graphene membrane at short and long oxidation periods are electrostatic repulsion and streric size exclusion, respectively. Graphene oxide (GO), chemically converted from graphene nanosheets, has oxygen functional groups such as hydroxyl and epoxy which enable it to have better water dispersibility than graphene [133,134]. Nair et al. invented a GO membrane consisting of closed-packed GO sheets that only allow water molecules to travel through and concurrently hinder the motion of other species [135]. Similarly, Zhao et al. designed a free-standing GO membrane in which the GO sheets are crosslinked by Ca^2+^ from Congo red dye [136]. More specifically, this GO membrane with tunable interlayer spacing was prepared by facile and thermal reduction methods using hot pressing method. Accompanied by relative high water permeability (17.1 LMH/bar), the resulting membrane showed excellent removal rates for heavy metal ions (i.e., 98.6% for Cu^2+^, 97.2% for Pb^2+^, 99.1% for Cd^2+^ and 97.2% for Ni^2+^). Although there have been many breakthroughs and exciting achievements for porous graphene and GO membranes in water filtration, special synthesis techniques for large-area porous membranes and fabrication reproducibility remain challenges towards commercialization.

### 2.3. Mixed Matrix Membranes

Mixed matrix membranes (MMMs), a currently popular area of research, are made by incorporating inorganic fillers into organic matrices. Although TFC membranes have excellent salt removal performance, there is a trade-off between permeability and selectivity. The main advantage of MMMs is to combine the low manufacturing cost, outstanding selectivity and high packing density of polymeric materials with long-term stabilities, high mechanical strength and regeneration capability of ceramic materials. One type of MMMs is a polymeric membrane blended with inorganic nanoparticles, which can be prepared by dispersion crosslinking, interfacial polymerization, or dip coating. Inorganic fillers that have been investigated for this purpose include titania, zeolite, silica, alumina, etc., and experimental results indicate the addition of inorganic nanoparticles alter the polymeric structures and effect the transportation of molecules through membrane pores [137,138,139,140,141]. Therefore it is not surprising that small inorganic nanoparticles would improve the water purification performance of organic membranes. Titania is widely used in anti-fouling coating due to its photocatalytic property. Kim et al. studied the influence of TiO_2_ fillers on the properties of carboxylate groups functionalized TFC membranes and found the carboxylate groups help the adsorption of titania on TFC membrane surface, which result in very good anti-biofouling properties, especially under UV excitation [142]. Such a hybrid RO membrane also has stable surface structure since no significant loss of titania particles was observed after being tested for 168 h [143]. Researchers also recognized the addition of zeolite and silica nanoparticles increases the surface roughness, contact angle, and water flux [144,145]. NaA zeolite nanoparticles are the first successfully synthesized zeolite particles with low contact angle (<5°) and RO ranged pores (~0.5 nm) [146]. MMMs prepared with NaA zeolite fillers by interfacial polymerization method have many outstanding properties, that is, more negatively charged and hydrophilic surface with increasing zeolite content, enhanced water permeability, and better water purification performance [147].

Composite membrane synthesized from carbon-based materials and organic materials is another type of MMMs. Majumder et al. reported a polystyrene membrane incorporated with MWCNTs which have an average diameter of 7 nm [148]. The MWCNTs were grown and aligned by catalytic chemical vapor deposition (cCVD) method, followed by spin coated on polystyrene matrix to seal gaps between CNTs. The tips of MWCNTs were opened by plasma etching approach, and the water flux of the synthesized composite membrane was 4–5 orders of magnitude higher than that calculated from Hagen-Poiseuille theory, indicating macroscale hydrologic mechanism. On the other hand, some researchers explained the ultra-high water flux was due to the formation of a layer of water molecules along MWCNTs walls, which reduce the friction significantly when bulk mater molecules come through [149]. Furthermore, to simplify the complex fabrication steps of MMMs, a patient has been published recently about dispersing 0.8 nm diameter CNTs into cross-linking solutions during the formation of polymeric membranes, so that the CNTs can be embedded into the organic barrier layer on top of microporous polyethersulfone support [150]. After being functionalized by octadecylamine, tests were performed on membranes made with and without CNTs to demonstrate the improved water flux generated by CNTs pathways. Experimental results showed the flux of membrane containing CNTs was approximately twice as much as that without CNTs (44 L m^−2^ day^−1^ bar^−1^ compared with 26 L m^−2^ day^−1^ bar^−1^), and MMMs with CNTs also had a slightly better salt rejection (97.7% compared with 96.2%). Even though MMMs combine the benefits of both polymeric and ceramic membranes, they are difficult to study since the interface between various materials may have unwanted structure and certain great materials become insoluble in each other. In addition, studies on MMMs with larger surface area are necessary before developing manufacturing apparatus for large-scale production.

## 3. Challenges and Future Perspectives

Although the water purification market has been occupied by polymeric membranes for more than 10 years, research and development activities in polymeric membranes are reaching the bottleneck and many industries still use traditional TFC membranes such as PA membrane which was introduced nearly 40 years ago. Despite expansions of TFC membranes and related techniques, it is time to upgrade RO technology to a new height or develop another cutting-edge technology for water purification. Addition of functional materials such as inorganic fillers, lyotropic crystals, CNTs, MWCNTs, and aquaporins can optimize the water flux and/or salt rejection, but the high cost issue associated with synthesis and blending these materials needs to be addressed before scale-up production and commercialization [151,152]. Meanwhile, new models are needed to predict the performance of composite membranes. Traditional polymeric RO and NF membranes are commonly modeled based on extended Nernst-Planck equation, which needs to be modified for carbon-based MMMs [153]. Recent models applied to calculate water flux and salt rejection of charged membranes for aqueous electrolyte solutions are listed in Table 5. For organic membranes blended with CNTs, CNTs can be simplified as circular cylinders, the fluid transport of which can be modeled using Hagen-Poiseuille equation. The flow through pores outside the CNTs and within the polymeric matrix can still be studied by extended Nernst-Planck model concerning dielectric exclusion since the dielectric constants for feed water, CNTs and organic matrix are different and electrostatic interactions will happen between ions in feed solution and polarization charges formed along the boundary of various dielectric media [154]. Assuming that the CNTs are distributed uniformly in polymeric base, the predicted model for such MMMs is likely to be extended Nernst-Planck formula plus an additional Hagen-Poiseuille term. Both terms are re-written according to their corresponding concentration before addition. The modeling of MMMs with GO fillers is more complicated and depends on the insertion direction: if GO is blended vertically into organic membrane like CNTs, similar equation of CNTs MMMs can be used for GO MMMs; If GO is added horizontally, the tortuosity factor in the extended Nernst-Planck equation needs to be revised due to the fact that the ion transport path inside GO is different from that in polymeric matrix. Additionally, since the functional groups located on the surface of GO (types of functional groups are determined by synthesis method, precursor materials, etc.) can react with ions in fluid and form complexes, the flux and permeability may change with time, indicating possible process-model mismatch. On the other hand, advanced techniques including rapid thermal processing (RTP) and nanorods fabrication enable the generation of defect-free membranes for water treatment applications. In addition to the use of new materials and leading-edge technologies, membrane diameter also plays an important role in enhancing filtration performance. Membranes with large surface area could reduce capital cost and energy consumption by approximately 15% [68]. Furthermore, different water treatment plants have specific difficulties to overcome. For instance, low recovery rate of seawater, disposal of brine and high capital cost are the biggest challenges that nowadays desalination plants confront. Tarquim et al. have developed a method to minimize produced brines, which results in good recovery rate, but more research and equipment are needed to reduce brine disposal [155]. Moreover, integration of traditional synthesis process with renewable energy may make green fabrication of nanocomposite membranes possible.

The excellent filtration performance of inorganic membranes, as stated in Table 4, indicates the capacity of ceramic membranes for most water purification applications, and the low acceptance of inorganic membranes in the past is because of the sheer dominance of polymeric RO and NF membranes in large-scale water treatment systems. Recent research on preparation of advanced inorganic membranes such as free-standing CNTs membranes and interlayer free membranes enables efficient filtration process with better purification performance and lower facility cost [8,162]. According to Weschenfelder et al., the operation expense and total cost of a water treatment plant using ceramic membranes with a flow rate of 2 m/s and water recovery rate of 95% are US $0.23/m^3^ and US $3.21/m^3^, respectively [163]. Similar to polymeric membranes, the development and manufacturing costs of ceramic membranes remains a significant problem for their industrialization. For example, although there have been rapid growth and development for CNTs and MWCNTs membranes in laboratory-scale, the commercial applications of carbon-based membranes are ongoing in a low pace due to the high cost of synthesizing CNTs and MWCNTs. Thanks to recent advancements in fabrication technology including cCVD, large-scale synthesis of high-quality CNTs economically is achievable. However, the reproducibility and feasibility of these methods for making membranes are in doubt. For traditional metal oxide membranes, high cost of supports is a challenging issue for commercialization. Current research focuses on studying alternative inorganic membranes made from cheaper or waste materials such as coal fly ash to reduce the manufacturing investment.

## 4. Conclusions

Tremendous amount of effort has been made to overcome the clean water scarcity and nanotechnology is a strong candidate with fast development. Study and commercialization of polymeric RO and NF membranes started in the early 1960s. So far the water desalination market is dominated by two kinds of membranes: cellulose-based (CA) membranes and thin-film composite (TFC) membranes. The most representative products such as TS40, TS80 and AD-90 were developed more than 30 years ago and due to their low manufacturing costs and high salt rejections, no major change has been made since then. New research directions for barrier layers in TFC membranes include improvement of fouling resistance as well as chemical and thermal stabilities. Meanwhile microporous supports can be optimized to increase the mechanical strength and permeability.

Inorganic RO and NF membranes have been studied in lab scale for water purification since the 1980s. The most representative ceramic membranes are metal oxide membranes and carbon-based membranes. The main synthesis method for metal oxide membranes is sol-gel technique, which needs further optimization to control the particle size and distribution. The performance of mixed matrix membranes (MMMs) made with both organic and inorganic nanomaterials is excellent, yet they are too expensive compared with other membranes. Hence it is important to realize the economic competitiveness of MMMs, as well as their potential applications. While nanotechnology is leading the way in developing RO and NF membranes for water purification, there are still technical and scientific problems that need to be solved before more benefits can be realized. Despite the challenges to be overcome, it is highly possible that ceramic membranes will be commercialized and industrialized in water purification and desalination fields in the near future.

## Figures and Tables

**Table 1:** Commercial polymeric RO and NF membranes for water purification.

Membrane	Manufacturer	Selective Layer	Maximum Temperature (°C)	pH Range	Salt Rejection (%)
SW30HRLE-400	Dow Filmtec, USA	PA TFC	45	2–11	99.8 NaCl
NF270-400/34i	Dow Filmtec, USA	PA TFC	45	3–10	>97 NaCl
SWC4+	Hydranautics, USA	PA TFC	45	3–10	>99.7 NaCl
TM820C-370	Toray, USA	PA TFC	45	2–11	>99.5 NaCl
HB10255	Toyobo, Japan	CTA hollow fiber	40	3–8	>99.4 NaCl
TS40	Microdyn-Nadir, USA	Polypiperazineamide	45	1–12	40 NaCl >98.5 MgSO_4_
TS80	Microdyn-Nadir, USA	PA TFC	45	1–12	80 NaCl >98.5 MgSO_4_
AD-90	GE-Osmonics, USA	TFC	50	4–11	>99.5 NaCl 95% Boron
AG4040C	GE-Osmonics, USA	TFC	50	4–11	>99 NaCl
HL2540FM	GE-Osmonics, USA	TFC	50	3–9	>96 MgSO_4_
CK4040FM	GE-Osmonics, USA	CA	30	5–6.5	>94 MgSO_4_
8040-SW-400-34	Koch, USA	Proprietary PA TFC	45	4–11	>99.5 NaCl
4040-HR	Koch, USA	Proprietary PA TFC	45	4–11	>99.2 NaCl
MPS-34 2540 A2X	Koch, USA	Proprietary composite NF	50	0–14	35 NaCl 95 Glucose 97 Sucrose
NFX	Synder, USA	Proprietary PA TFC	50	2–11	40 NaCl >99 MgSO_4_ >99 Lactose
NFW	Synder, USA	Proprietary PA TFC	50	2–11	20 NaCl >97 MgSO_4_ >98.5 Lactose

**Table 2:** Effects of various processing methods on chlorine resistance.

Membrane	Processing Method	Performance Evaluation	Reference
Cellulose acetate	Blending with polyethersulfone and polyethylene glycol	Such blended membranes had higher porosity (permeability) and chlorine tolerance compared with virgin cellulose acetate membranes.	[36]
Sulfonated poly	Made with high fluorine contents	Sulfonated-fluorinated poly membranes displayed long-term stability (>30 days) under high acidic chlorine condition.	[37]
Aromatic polyamide	Adding 0.1–1 wt% multi-walled carbon nanotubes	The carbon nanotube based polyamide membranes had good selectivity and longer lifetime during desalination process.	[38]
Sulfonated poly	Membranes were prepared by direct copolymerization method	Water permeability and contact angle remained unaffected when exposed to high level of chlorine and wide range of pH (4–10).	[39]
Cellulose triacetate	Adding sodium hexametaphosphate (SHMP) as masking agent	SHMP inhibited oxidation degradation of cellulose triacetate membranes by chlorine.	[40]
Sulfonated cardo poly	Extra layer of formaldehyde-cross-linked polyvinyl alcohol was coated on membrane surface	The coated layer improved NaCl rejection from 91.2% to 96.8% and the membrane showed better chlorine resistance in RO operation.	[41]
Polyamide	Membrane synthesized by interfacial polymerization of *N*-phenylethylenediamine and 1,3,5-benzenetricarbonyl trichloride	When immersed in NaOCl solution, the membrane exhibited higher chlorine tolerance than a commercial polyamide membrane.	[42]

**Table 3:** Monomers and performance evaluation for thin-film composite (TFC) membranes prepared by interfacial polymerization method.

Monomer A	Monomer B	Performance Evaluation	Reference
Ethylenediamine	Cyclodextrins	Membrane had a water flux up to 28 L/m^2^ h (LMH) and good antifouling properties with flux reduction <20%.	[75]
Piperazine	1,3,5-Benzene-tricarbonyl trichoride	High salt rejection (98% for Na_2_SO_4_ and 97.5% for MgSO_4_) with enhanced water permeability.	[76]
*m*-Phenylenediamine	Trimesoyl chloride	Membrane exhibited large free volume, high water flux, and low reverse salt flux.	[77]
Hexylene glycol	1,3,5-Benzene-tricarbonyl trichoride	Both flux stability and fouling reversibility improved for Ca^2+^ modified membranes.	[78]
1,3-Phenylenediamine	1,3,5-Benzene-tricarbonyl trichoride	Membranes with two PA layers showed much higher flux and selectivity than commercial TFC membranes.	[79]
Piperazine	2,4,6-Trischlorosulfonylphenol	Membrane had a flux of 13.98 LMH and good rejections for CuSO_4_ and H_2_SO_4_.	[80]
Polyallylamine	1,3-Benzenedisulfonyl chloride	Membrane was positively charged and had selectivities greater than 90% for heavy metal ions.	[81]
*p*-Phenylenediamine	1,3,5-Triformylphloroglucinol	Membrane presented a stable rejection to Congo red of 99.5% and a high flux up to 50 LMH.	[82]
*n*-Aminoethyl piperazine propane sulfonate	Trimesoyl chloride	Compared with pristine membrane, the flux increased by 82% while the NaCl rejection remained above 98%.	[83]
Pentaerythritol	Trimesoyl chloride	Membrane had a high rejection of Na_2_SO_4_ (98.1%) but a low water flux of 6.1 LMH.	[84]

**Table 4:** State-of-the-art inorganic RO and NF membranes for water purification.

Membrane.	Application	Salt Rejection (%)	Flux/Permeability	Reference
γ-Al_2_O_3_	Desalination	97.1 Fe^3+^, 90.9 Al^3+^, 85 Mg^2+^, 84.1 Ca^2+^, 30.7 Na^+^, 27.3 NH_4_^+^	17.4 LMH/bar	[85]
PVA-Al_2_O_3_	Dye wastewater treatment, Desalination	96 Congo red dye 3 NaCl	25 LMH	[86]
CMS-Al_2_O_3_	Desalination	93 NaCl	25 kg m^−2^ h^−1^, 3.5 wt% NaCl, 75 °C	[87]
Al_2_O_3_ (FAS grafted)	Desalination	>99.5 NaCl	19.1 LMH, 2 wt% NaCl, 80 °C	[88]
TiO_2_	Desalination	99 NaCl	6 kg m^−2^ h^−1^, 10 wt% NaCl, 75 °C	[89]
ZrO_2_	High salinity water treatment	>90 PEG 1000 68, 24.92 wt% NaCl	13 LMH/bar	[90]
TiO_2_-ZrO_2_	Radioactive waste treatment	99.6 Co^2+^, 99.2 Sr^2+^, 75.5 Cs^+^	40 LMH/bar	[91]
SiO_2_	Desalination	99.5 NaCl	6.6 kg m^−2^ h^−1^, 3.5 wt% NaCl, 22 °C	[92]
SiO_2_	Desalination	99.6 NaCl	9.5 kg m^−2^ h^−1^, 3.5 wt% NaCl, 22 °C	[93]
CoO-SiO_2_	Desalination	99.7 NaCl	7.7 kg m^−2^ h^−1^, 3.5 wt% NaCl, 22 °C	[94]
Ax-GO	Desalination	99.9 NaCl	19.7 kg m^−2^ h^−1^, 3.5 wt% NaCl, 90 °C	[95]
CNT-rGO	Drinking water purification	97.3 Methyl orange	20–30 LMH/bar	[96]
TiO_2_-GO	Dye wastewater treatment	>97 Organic dyes	89.6 LMH/bar	[97]
APT-GO	Dye wastewater treatment	~100 Rhodamine blue	13.3 LMH, 7.5 mg L^−1^ RhB	[98]
MoS_2_	Dye wastewater treatment	100 Methylene blue	135.3 LMH/bar	[99]
YSZ	Dye wastewater treatment	>98 NaCl	28 LMH/bar	[100]

**Table 5:** Recent models for transport of aqueous electrolytes through charged membranes.

Suitable Retention Mechanisms	Model	Model Evaluation	Reference
UF	Irreversible thermodynamic model	The model can be used to predict the performance for single electrolyte solution but not for mixed electrolyte solutions.	[156]
RO/UF	Extended Nernst-Planck model	Single-ion rejection calculated from the model matched with that obtained from irreversible thermodynamic model, and there is little difference between mixed-ion rejection and experimental data.	[153]
NF	Solution-diffusion-electromigration model	Easily modeled chloride and sulfate selectivities with transmission coefficient simplified to zero.	[157]
RO	Merten and Lonsdale transport model	The model gave concentration polarization corrected salt transport coefficients whose effects were significant at high feed pressures.	[158]
RO/NF	Donnan steric pore model and dielectric exclusion	Dielectric exclusion was considered as the primary effect when analyzed mass transfer of electrolytes and neutral solutes.	[159]
NF	Coupled series-parallel resistance model	This model was developed specifically for organic solvents permeating through ceramic membranes and a good fit to experimental data was obtained for different solvents.	[160]
RO/NF	Pore blockage-cake filtration model	Model had similar results and coefficient of determination as Faridirad model, but with lower Akaike information criteria values.	[161]
